# Clinical evaluation of a 31‐year‐old woman with crural monomelic amyotrophy

**DOI:** 10.1002/ccr3.2557

**Published:** 2019-12-03

**Authors:** Austin R. Thompson, Brooke Beckett, Erik R. Ensrud

**Affiliations:** ^1^ Department of Orthopaedics and Rehabilitation Oregon Health & Science University Portland Oregon; ^2^ Department of Diagnostic Radiology Oregon Health & Science University Portland Oregon

**Keywords:** crural, crural monomelic amyotrophy, electromyography, magnetic resonance imaging, monomelic amyotrophy, neurogenic muscular atrophy

## Abstract

This report describes the clinical presentation of a female patient diagnosed with crural MMA. Careful clinical correlation is necessary to distinguish crural MMA from other motor neuron diseases. When crural MMA is diagnosed, treatment options aim to alleviate symptoms.

## Graphical abstract

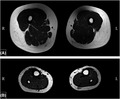

## 1 INTRODUCTION

Crural monomelic amyotrophy is an uncommon motor neuron disease with muscle atrophy and weakness affecting one lower limb. This report presents a delayed diagnosis about 10 years after the initial onset of symptoms and describes the clinical evaluation and treatment options for crural monomelic amyotrophy.

Monomelic amyotrophy (MMA) is an uncommon disorder characterized by neurogenic atrophy restricted to a single upper (Hirayama disease) or lower (crural) limb. The exact etiology of MMA is unknown.^1^ Both brachial and crural MMA have similar clinical presentations, with most cases being reported in Asian countries, in the second or third decade of life, with a higher prevalence in males, and with weakness and atrophy in a single limb.^1^ Clinical evaluation includes physical examination, electromyography (EMG), magnetic resonance imaging (MRI), laboratory testing, or muscle biopsy.^2^

There are signs and symptoms only specific to crural MMA.^1^ Involvement of the intrinsic foot muscles is less frequent than leg or thigh muscles. Foot drop may be present, with patients reporting minimal disability and exertion‐induced fatigue. MRI may help determine specific muscle involvement.^3^, ^4^ While possible theories regarding the etiology of crural MMA have been discussed,^1^, ^2^, ^3^, ^4^ we aim to describe the clinical presentation of our patient and describe treatment options to alleviate the signs and symptoms of crural MMA.

## 2 CASE PRESENTATION

The patient is a Caucasian woman who developed slowly progressive left leg atrophy around age 20. She was diagnosed with morphea in the left mid abdomen at age 12, which remained localized. She has been previously evaluated by multiple specialties (podiatry, neurology, dermatology, and rheumatology) for her left foot problems since the onset, with no diagnosis made of the underlying pathology. Bilateral foot radiographs showed less proximal left foot soft tissue compared with the right. Lumbar spine MRI did not show significant neural foraminal stenosis or spinal canal stenosis. A prior EMG of the left lower extremity was reported as normal at 28‐years‐old, as was a Doppler ultrasonography study. Laboratory results were normal for serum chemistries, anemia, thyroid hormones, serum Lyme titer, and autonuclear antibodies. Creatine kinase (CK) was 81 U/L.

At 31 years old, she was referred to our clinic for left leg atrophy. She reported that she had been experiencing left leg problems with a slow, progressive course which became stable weakness. She stated that the symptoms are located in the distal left leg, which started as atrophy of the intrinsic foot and progressed to the anterior/posterior calf muscles and mild atrophy of the left thigh. She described a sharp, achy pain on the left plantar foot that was exacerbated with weight bearing. She reported occasional exercise‐induced cramps in the affected limb, without numbness or tingling in the affected limb. She reported that symptoms improve with the use of a custom‐molded orthotic and gabapentin.

Physical examination revealed Medical Research Council (MRC) scores of 4+/5 left knee extension, 4−/5 left knee flexion, and 4+/5 left ankle dorsiflexion. She had difficulty completing a single leg standing toe raise on her left leg. All other manual muscle tests and reflexes were normal. No scapular winging was present. Circumference measurements for the right and left thigh 5 cm above the patella were 39.0 cm and 34.0 cm, respectively. The circumference of the right and left leg at 5 cm below the tibial tuberosity was 33.5 cm and 30.5 cm, respectively. An EMG of the bilateral lower extremity and MRI of the bilateral femur and tibia/fibula were ordered.

**Figure 1:**
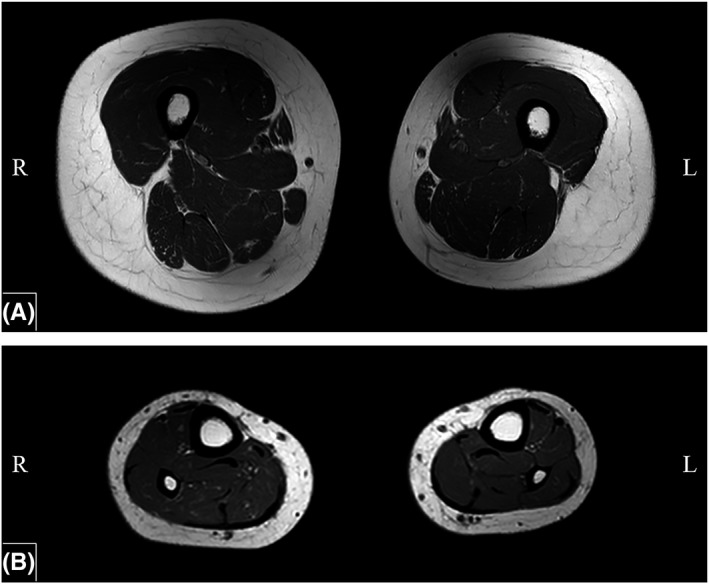
A, Bilateral Femur T1W Axial MRI without contrast. B, Bilateral Tibia/Fibula T1W Axial MRI without contrast

The EMG showed evidence of mild reinnervation changes without acute denervation in the left lower extremity. The MRI of the thighs showed minimal subjective decreased left muscle bulk, without muscle edema (Figure 1A). The MRI of the lower legs showed mild edema within the left flexor digitorum longus, flexor halluces longus, and tibialis anterior, with slightly increased asymmetric volume loss of the left flexors (Figure 1B). No left foot muscle atrophy, decreased muscle bulk, or muscle edema was noted.

A 10 mm deep punch biopsy of the left anterior thigh showed did not identify morphea. Two additional biopsies of the left anterior thigh were performed: (a) superficial and (b) deep fat and fascia. However, there was not significant sclerosis upon which to base a diagnosis of morphea.

Findings from the medical history, physical examination, and diagnostic tests were consistent with crural MMA. The diagnosis was made approximately 10 years after the patient reported the first symptoms. She was referred for a custom ankle‐foot orthosis to assist in lengthy hikes and reduce left foot pain.

## 3 DISCUSSION

This report describes a case of Caucasian woman diagnosed with crural MMA approximately 10 years after the onset of symptoms. MMAs are uncommon neuromuscular disorders, with crural MMA less common than brachial MMA. Nalini et al present and comprehensively describe clinical features of the largest series of patients with MMA; of 279 patients in the series, 55 had crural MMA.^1^ However, the vast majority of crural MMA cases have been reported in men from Asian countries. To our knowledge, crural MMA has not previously been reported in a Caucasian women.

Given prior evaluations by multiple specialties, a wide variety of other disorders were excluded from the differential diagnosis. With symptoms presenting in only the left lower extremity since the onset and no upper motor neuron symptoms or signs, amyotrophic lateral sclerosis was unlikely.^5^ Multifocal motor neuropathy was also considered. However, there was no conduction block identified on the EMG or other relevant laboratory findings.^6^ Facioscapulohumeral muscular dystrophy can also present with isolated monomelic lower limb atrophy with elevated CKs and normal thigh MRIs.^7^ Neither the patient nor her family members reported weakness in facial or periscapular muscles. Additionally, the muscle atrophy extended into the thigh, and the patient did not have abnormal CK values. With the patient's history of morphea, there was clinical concern to rule out the soft tissue atrophy as an expansion of the abdominal spot. Furthermore, morphea profunda has been described to mimic neuromuscular disease since it can cause progressive muscle atrophy.^8^ While morphea profunda may present in different anatomical locations than skin lesions from cutaneous morphea, multiple biopsies using MRI to identify biopsy sites were not diagnostic of morphea. Our patient also had neurogenic findings upon EMG examination, which has not been observed in cases of morphea profunda.^8^

While there are potential definitive treatment options for patients with more common motor neuron diseases, treatments for patients with crural MMA are not well described. Clinicians may reduce functional deficits and pain through different interventions. Physical therapy protocols can assist with limb strength and joint range of motion. Ankle‐foot orthoses and other bracing options can improve physical function by reducing the effects of weak stabilizing muscles. Gabapentin may reduce pain and spasticity in patients with motor neuron diseases.^9^ Mindfulness training is another option to alleviate some of the symptoms.^10^ Although many different forms of mindfulness training exist, its reliably improves pain catastrophizing, anxiety, and depression. In combination, these treatments may reduce both the psychological and physical symptoms of crural MMA.

This report describes the clinical presentation of a female patient diagnosed with crural MMA. Careful clinical correlation is necessary to distinguish crural MMA from other motor neuron diseases. When crural MMA is diagnosed, treatment options aim to alleviate symptoms.

## CONFLICT OF INTEREST

None of the authors have any conflict of interest to disclose.

## AUTHOR CONTRIBUTIONS

AT: conducted the literature search on the topic and drafted the initial version of the manuscript. BB: reported clinical information and provided critical revision of the manuscript for intellectual content. EE: collected clinical information and provided critical revision of the manuscript for intellectual content.
